# Probiotic treatment with viable α‐galactosylceramide‐producing *Bacteroides fragilis* reduces diabetes incidence in female nonobese diabetic mice

**DOI:** 10.1111/1753-0407.13593

**Published:** 2024-08-13

**Authors:** Camilla H. F. Hansen, Danica Jozipovic, Line F. Zachariassen, Dennis S. Nielsen, Axel K. Hansen, Karsten Buschard

**Affiliations:** ^1^ Department of Veterinary and Animal Sciences, Faculty of Health and Medical Sciences University of Copenhagen Frederiksberg C Denmark; ^2^ Department of Food Science, Faculty of Science University of Copenhagen Frederiksberg C Denmark; ^3^ Department of Pathology The Bartholin Institute, Rigshospitalet Copenhagen Denmark

**Keywords:** alpha‐galactosylceramide, *Bacteroides fragilis*, gut barrier, M2 macrophages, NKT cells, type 1 diabetes

## Abstract

**Background:**

We aimed to investigate whether alpha‐galactosylceramide (α‐GalCer)‐producing *Bacteroides fragilis* could induce natural killer T (NKT) cells in nonobese diabetic (NOD) mice and reduce their diabetes incidence.

**Methods:**

Five‐week‐old female NOD mice were treated orally with *B. fragilis*, and islet pathology and diabetes onset were monitored. Immune responses were analyzed by flow cytometry and multiplex technology. Effects of ultraviolet (UV)‐killed α‐GalCer‐producing *B. fragilis* and their culture medium on invariant NKT (iNKT) cells were tested ex vivo on murine splenocytes, and the immunosuppressive capacity of splenocytes from *B. fragilis*‐treated NOD mice were tested by adoptive transfer to nonobese diabetic/severe combined immunodeficiency (NOD/SCID) mice.

**Results:**

*B. fragilis* reduced the diabetes incidence from 69% to 33% and the percent of islets with insulitis from 40% to 7%, which doubled the serum insulin level compared with the vehicle‐treated control mice. Furthermore, the early treatment reduced proinflammatory mediators in the serum, whereas the proportion of CD4^+^ NKT cell population was increased by 33%. *B. fragilis* growth media stimulated iNKT cells and anti‐inflammatory M2 macrophages ex vivo in contrast to UV‐killed bacteria, which had no effect, strongly indicating an α‐GalCer‐mediated effect. Adoptive transfer of splenocytes from *B. fragilis*‐treated NOD mice induced a similar diabetes incidence as splenocytes from untreated NOD mice.

**Conclusions:**

*B. fragilis* induced iNKT cells and M2 macrophages and reduced type 1 diabetes in NOD mice. The protective effect seemed to be more centered on gut–pancreas interactions rather than a systemic immunosuppression. *B. fragilis* should be considered for probiotic use in individuals at risk of developing type 1 diabetes.

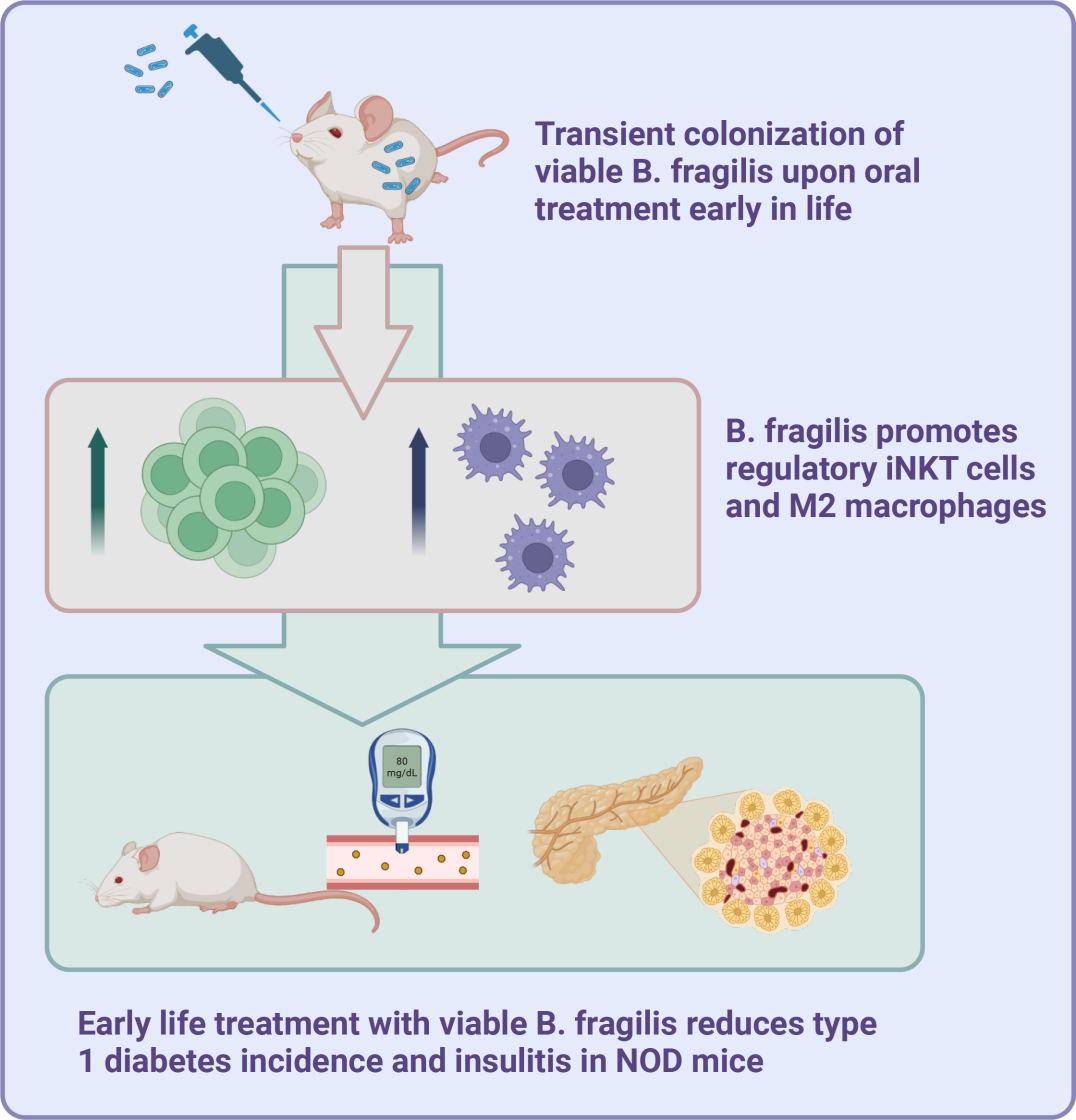

## Graphical abstract

Highlights

- Transient colonization by viable *B. fragilis* reduces type 1 diabetes incidence in NOD mice.
- Alpha‐galactosylceramide‐producing *B. fragilis* induces anti‐inflammatory immunity.
- Protection against diabetes by *B. fragilis* is not based on systemic immune cells.
- *B. fragilis* may become a next‐generation probiotic for prevention of type 1 diabetes.

## 1 INTRODUCTION

Alpha‐galactosylceramide (α‐GalCer) is a glycosphingolipid found on a marine plant in the Japanese sea.^1^ It is a potent and specific activator of natural killer T (NKT) cells, a unique subset of cells important for regulating the immune system.^2^ Invariant NKT cells (iNKT or type 1 NKT cells) express an invariant T‐cell receptor and are defined by their reactivity to α‐GalCer after loading to cluster of differentiation (CD) 1d molecules. However, for a long time, the isolation of a mammalian antigen that was able to activate iNKT cells continuously failed as all mammalian sphingolipids are in β‐glycosphingolipid form.^3^, ^4^ It has now been found that certain *Bacteroides* spp. that reside in the mammalian intestines can produce α‐GalCer.^5^ These Gram‐negative obligate anaerobic bacteria can induce iNKT cells when presented by CD1d on antigen‐presenting cells in mice and humans.^5^ iNKT cells produce interferon (IFN)‐γ and interleukin (IL)‐4 and, in particular, T helper (Th) 2 cytokines upon repeated activation.^6^ The involvement of iNKT cells in type 1 diabetes was first highlighted by the observations that qualitative and quantitative defects in iNKT cells exist in nonobese diabetic (NOD) mice,^7^ and later confirmed in both CD1d‐deficient NOD mice^8^, ^9^, ^10^ and by adoptive transfer experiments of iNKT cells, which accelerated and prevented type 1 diabetes, respectively.^11^, ^12^ Injection of α‐GalCer‐ or α‐GalCer‐activated iNKT cells has demonstrated the protective mechanisms to be dependent on IL‐4‐ and regulatory T cell (Treg)‐dependent mechanism^8^, ^13^, ^14^ and recruitment of tolerogenic myeloid dendritic cells.^10^ As a lower abundance of *B. fragilis* is found in human type 1 diabetes patients compared with healthy controls,^15^ we hypothesized that the intestinal commensal *B. fragilis* plays an important role in suppressing type 1 diabetes when given as a probiotic to NOD mice by activating NKT cell‐mediated immunoregulatory functions.

## 2 METHODS

### 2.1 Ethics

The experiment complied with the Danish *Act on Animal Experimentation* which implements the Directive 2010/63/EU on the Protection of Animals used for Scientific Purposes. It was approved by the Danish Animal Experiments Inspectorate at Ministry of Food, Agriculture, and Fisheries of Denmark (License number: 2017‐15‐0201‐01262).

### 2.2 Animal study design

Four‐week‐old NOD/MrkTac female mice from Taconic (Germantown, NY) were group‐housed (five mice/cage) as previously described.^16^ From 5 until 8 weeks of age, the mice were oral gavaged with either 100 μL (10^9^ colony forming units (CFU) total) *B. fragilis* NCTC9343 or vehicle three times per week (nine times total) based on randomized allocation to groups. At least four cages were included per group. Anaerobe basal broth (M1636, Himedia, Maharashtra, India) was used as growth media for the bacteria under anaerobic conditions for 48 h, before it was centrifuged and dissolved in saline peptone water for oral gavage of mice. Mice were euthanized when diagnosed as diabetic or at 30 weeks of age. Measurements of tail blood glucose commenced twice a week from 12 weeks of age, and a mouse was considered diabetic when blood glucose levels exceeded 12 mmol/L on two consecutive days. Body weight of all offspring was monitored once a week. Additional NOD mice were similarly treated (*n* = 2–3 per cage) and euthanized the day the treatment ended at 8 weeks of age. The spleen was used for adoptive transfer, by aseptic intraperitoneal (IP) injection of 20 million splenocytes into 8‐weeks‐old female NOD/MrkBomTac‐*Prkdc* ^*scid*^ mice (Taconic). The nonobese diabetic/severe combined immunodeficiency (NOD/SCID) mice were monitored for diabetes by weekly blood glucose measurements for 24 weeks.

### 2.3 Histology of pancreas

At 8 weeks of age, insulitis was scored in the pancreas of the nondiabetic donor mice for the adoptive transfer experiment in a blinded fashion on 30 islets per pancreas on hematoxylin and eosin (H&E)‐stained 5 μm sections that were cut with 100 μm interval and were graded as previously described.^17^

### 2.4 Serum endocrine and inflammatory markers

Serum levels of IFN‐γ, IL‐1β, IL‐2, IL‐4, IL‐5, IL‐6, C‐X‐C motif ligand 1 (CXCL1), IL‐10, IL‐12p70, and tumor necrosis factor (TNF)‐α were measured in 30‐week‐old nondiabetic mice using V‐PLEX Proinflammatory Panel 1 Mouse kit (Meso Scale Diagnostics, Rockville, MD) on a MESO QuickPlex SQ120MM and analyzed by the standard software Discovery Workbench (MSD).

Serum insulin in the 8‐week‐old adoptive transfer donor mice was measured with a Mouse Insulin ELISA kit (Mercodia, Uppsala, Sweden) following manufacturer's instructions. Function of β cells were assessed by calculating the homeostatic model assessment of β cell function (HOMA‐β) value based on blood glucose and insulin measurements by the following formula:

$$\text{HOMA}-\beta =\left(20\times \text{insulin}\left(\mathrm{\mu IU}/\mathrm{mL}\right)\right)/\left(\text{glucose}\left(\text{mmol}/L\right)-3.5\right)\%.$$

### 2.5 Fecal gut microbiota analysis

Feces were sampled before treatment at 5 weeks of age, during treatment at 8 weeks of age, and 3 weeks after treatment had ended at 11 weeks of age. DNA was extracted with QIAamp DNA stool mini kit (Qiagen, Hilden, Germany). Two primer sets, universal for the V3 region of the 16 s RNA gene,^18^ and *B. fragilis*‐specific PRBA388F (5′‐ACWCCTACGGGWGGCAGCAG‐3′) and PRUN518R (5′‐ATTACCGCGGCTGCTGG‐3′), and SYBR green polymerase chain reaction (PCR) master mix (Applied Biosystems, Foster City, CA) were used on a 7500 Fast real‐time PCR instrument (Applied Biosystem) with running conditions as previously described.^19^ *Escherichia coli* K‐12 was used as a positive control and standard, and quantified *B. fragilis* values were normalized to the 16S rRNA gene universal primer result. In addition, the samples from the 8‐week‐old mice, at the end of treatment, were used for 16S rRNA gene sequencing on the Oxford Nanopore long‐read sequencer MinION.

For this, all the samples were diluted to 5 ng/μL in order to be used in the first round of PCR (PCR1) library preparation. Furthermore, PCR1 ON_UMI16S library was prepared using 12 μL of PCRBIO Ultra Mix, 6 μL of sterile nuclease‐free MilliQ water, and 2 μL of primers mix (containing UMI_338ab_f/UMI_1391_R and UMI_27ab_F/UMI_1540_R of 5 μL) per a 5 μL of DNA sample (~5 ng/ μL) equaling in a total volume of 25 μL for each well. ThermoCycle machine values were 5 min denaturation at 95°C, followed by 2 cycles of 95°C for 20 s, 48°C for 30 s, 65°C for 10 s, 72°C for 45 s, and finally ending with elongation at 72°C for 4 min. PCR1 cleanup was conducted by transferring 10 μL Binding Beads solution that was previously suspended using Hula mixer and equilibrated to room temperature, to 25 μL of each PCR product. Once the content was mixed, the plate was placed into the magnetic rack, and supernatant was discharged. Binding Beads pellet were then washed with freshly prepared 80% ethanol twice, leaving the ethanol in the plate for 30 s before its removal. The plate was removed from the magnetic rack, and 15 μL of nuclease‐free MilliQ water was added to each well to resuspend the Binding Beads pellet. The final step of the PCR1 cleanup required placing the plate into the magnetic rack to remove the supernatant from the Binding Beads and transfer it to the protocol for the second round of PCR (PCR2). PCR2 reagents included 12 μL of PCRBIO Ultra Mix, 2 μL of ONT UMI barcodes (Set 1 was used), and 11 μL of the cleaned PCR1 product. ThermoCycle machine values were the following: denaturation at 95°C, followed by 33 cycles of 95°C for 20 s, 55°C for 20 s, 72°C for 40 s, and ending with elongation at 72°C for 4 min. After PCR2 ON_UMI, the gel electrophoresis was conducted to ensure the band size of ~1500 bp for PCR2 product. Gel validation was followed by pooling of the samples: 5 μL of each sample was pooled into one Eppendorf tube, including the positive, and excluding the negative control. Afterwards, the cleanup of the pooled PCR2 library was conducted by using resuspended UMI Beads. Then, 140 μL of UMI Beads was transferred to the Eppendorf tube containing 250 μL of the pooled samples. The mixture was incubated at room temperature for 5 min and then placed into the magnetic rack, followed by the removal of the clear colored supernatant. The procedure was followed by freshly prepared 80% ethanol wash twice. The beads were then resuspended with nuclease‐free MilliQ water, and the supernatant collected in a previously described manner. To ensure no primer‐dimer and a band size of ~1500 bp of the PCR2 cleaned library, the gel electrophoresis was performed. Finally, the concentration of the PCR2 pooled clean library was measured using the InvitrogenTM QubitTM4 Flourometer using the QubitR dsDNA HS Assay Kit (Excitation/Emission wavelength of 485/530 nm). The pooled barcoded amplicons underwent the Oxford Nanopore ligation protocol SQK‐LSK110 as a library preparation for MinION sequencing. Thereafter, 47 μL of DNA was used for end‐prep reaction mix. Finally, 75 μL of library was loaded on a R9.4.1 flow cell.

### 2.6 Gut microbiome analysis

The abundance table was generated from the data generated by MinION as previously described,^20^ and MicrobiomeAnalyst (https://www.microbiomeanalyst.ca/) was used for further analysis. Features having low counts (set to <2) and variance (10% removed based on inter‐quantile range) were removed during the filtration step. Two samples were excluded due to very low reads. All other samples were rarefied to even sequencing depth (10 829 reads), and total sum scaling was applied for normalization. Alpha (observed species index) and beta diversity (principal coordinate analysis (PCoA) based on Bray–Curtis and Jaccard distance matrices) were calculated. Permutational multivariate analysis of variance (PERMANOVA) was used to test differences between categories based on Bray–Curtis and Jaccard distance matrices. Analysis of compositions of microbiomes (ANCOM) was used to find compositional differences in the bacterial community.

### 2.7 Flow cytometry

Nondiabetic mice were euthanized at 30 weeks of age, and the pancreatic lymph node and spleen were immediately placed on ice. Antibody cocktails were used for staining NKT with CD3‐fluorescein isothiocyanate (FITC) labelled, NK/NKT‐phycoerythrin (PE) labelled, and CD4‐peridinin chlorophyll protein‐cyanine5.5 (PerCP‐Cy5.5) labelled; dendritic cells and macrophages with CD11c‐FITC, F4.80‐PE, and CD45 allophycocyanin (APC) labelled; and for intracellular staining of regulatory T cells with CD3‐FITC, forkhead box P3 (FoxP3)‐PE, and CD4‐PerCP‐Cy5.5 in single‐cell suspensions prepared, as previously described.^21^ All antibodies were purchased from eBioscience (San Diego, CA). Analysis was performed using an Accuri C6 flow cytometer (BD Biosciences, Franklin Lakes, NJ).

### 2.8 Ex vivo organ cell culture study design

Sterile single‐cell suspensions were made aseptically from spleen, mesenteric lymph node, and pancreatic lymph nodes from 6 to 7 NMRI mice (Taconic) in complete culture medium (RPMI 1640 supplemented with 10% fetal calf serum (FCS), 2 mmol/L L‐glutamine and 2 mmol/L penicillin and streptomycin; all from Sigma‐Aldrich, St. Louis, MO). The cells (5 × 10^5^ cells/well) were stimulated overnight (37°C for 18 h) with either clean growth media (0% or 33% of the total volume in the well), different doses of growth media in which *B. fragilis* was grown in and later separated from (1%, 7%, 17%, and 33% of the total volume), or ultraviolet (UV)‐killed bacteria in four different doses (3 × 10^3^, 3 × 10^4^, 3 × 10^5^, and 3 × 10^6^ CFU per well). Flow cytometric analysis of the cells was performed as described for the NOD mice with the antibody panel CD3‐FITC and α‐GalCer‐loaded CD1d tetramer (or unloaded control) for iNKT cells and F4.80‐FITC, CD86‐PE, CD11c‐PerCP‐Cy5.5, and CD45‐APC for macrophages and dendritic cells. All antibodies were from eBioscience except the tetramers which were a kind donation from National Institutes of Health (NIH) Tetramer Core Facility at Emory University (Atlanta, GA). The experiment was repeated three times with six to seven mice in each experiment, and statistical analyses on results from the ex vivo cell culture study are made with two‐way ANOVA based on time and treatment as factors and with Dunnett multiple comparison test. No significant differences were found between 0% or 50% clean media samples, and all post hoc statistical analyses were therefore made in comparison to a combined ‘clean media’ group as the control.

### 2.9 Arginase‐1 enzyme‐linked immunosorbent assay (ELISA)

Serum samples from the 30‐week‐old nondiabetic mice as well as supernatant from the ex vivo study, from the clean or *B. fragilis* growth media supplemented wells with highest doses, were used in a Mouse Arginase‐1 (Arg1) ELISA kit (Nordic Biosite, Täby, Sweden) performed according to manufactures manual. All samples were diluted 4 times. Absorbance was measured and analyzed on Epoch Bio‐Tek Instrument INC platereader (Winooski, VT).

### 2.10 Intestinal gene expression

Ileum and colon homogenate was prepared and RNA isolated with MagMAX‐96 RNA Isolation Kit (Applied Biosystems), and cDNA synthesis using High‐Capacity cDNA Reverse Transcriptase Kit (Applied Biosystems) was conducted as previously described.^22^ Specialized murine probes for immune and gut barrier related genes *Muc1*, *Muc2*, *Ocln*, *Tjp1*, *Cldn8*, *Cldn15*, *Pla2a2g*, *Ifng*, *Reg3g*, *Retn1d*, *Defa17*, *Cd8a*, *Tgfb*, *Prf1*, and *Gzmb*; (probe IDs: Mm00449604; Mm01276696, Mm00500912; Mm00493699; Mm00516972; Mm00517635; Mm00441127; Mm00445845; Mm04205962; Mm00842045; Mm00448160; Mm00475162; Mm00812512; Mm01168134; Mm00442834; all from Applied Biosystems) were used for quantitative polymerase chain reaction (qPCR). *Hprt* gene (probe ID: Mm03024075) was used as a reference gene. Samples were normalized to 3 ng/μL, and qPCR was conducted using a TaqMan assay mix containing 0.5 μL TaqMan Gene expression Assay (20×) (Applied Biosystems), 5 μL TaqMan Fast Universal PCR Master Mix (Applied Biosystems), and 2.5 μL Nuclease‐free water. Then, 2 μL of the sample was added to the total of 8 μL of the mix. Running conditions were first 20 s on 95C°, and then 39 cycles of 1 s at 95°C, followed by 20 s at 60°C on a Bio‐Rad C1000 Touch CFX96 Real‐Time System Thermal Cycler (Bio‐Rad, Hercules, CA). The amplification data were analyzed using the accompanying CFX Maestro software CFX Maestro (Bio‐Rad) to obtain threshold cycle (Ct) values. GenEx 6 (MultiD Analyses AB, Gothenburg, Sweden) was used for qPCR data transformation. The Genorm and Normfinder methods in GenEx were used to validate the stability of the selected reference gene, and the cycle threshold (Ct) values were normalized to the reference gene. For each gene, normalized expression levels were set relative to the sample with lowest expression to establish relative quantities (RQ) and were log2 transformed before statistical testing. False discovery rate was used to correct for multiple comparisons.

### 2.11 Statistics

GraphPad Prism version 8 (GraphPad Software, San Diego, CA) was used for statistical analysis; *p* values <0.05 were considered significant, but tendencies with *p* values <0.1 are shown. Cumulative diabetes incidence curves were calculated using the Kaplan–Meier estimation, while statistical significance between groups was evaluated by the log‐rank test. Other differences between the two groups were estimated by an unpaired two‐tailed Student's *t* test, or Mann–Whitney test if the data did not assume Gaussian distribution. Welch's correction was included if the *t* test if variances were unequal by the *F* test.

## 3 RESULTS

### 3.1 Early colonization of *B. fragilis* delays autoimmune diabetes onset and reduces the incidence later in life

**FIGURE 1:**
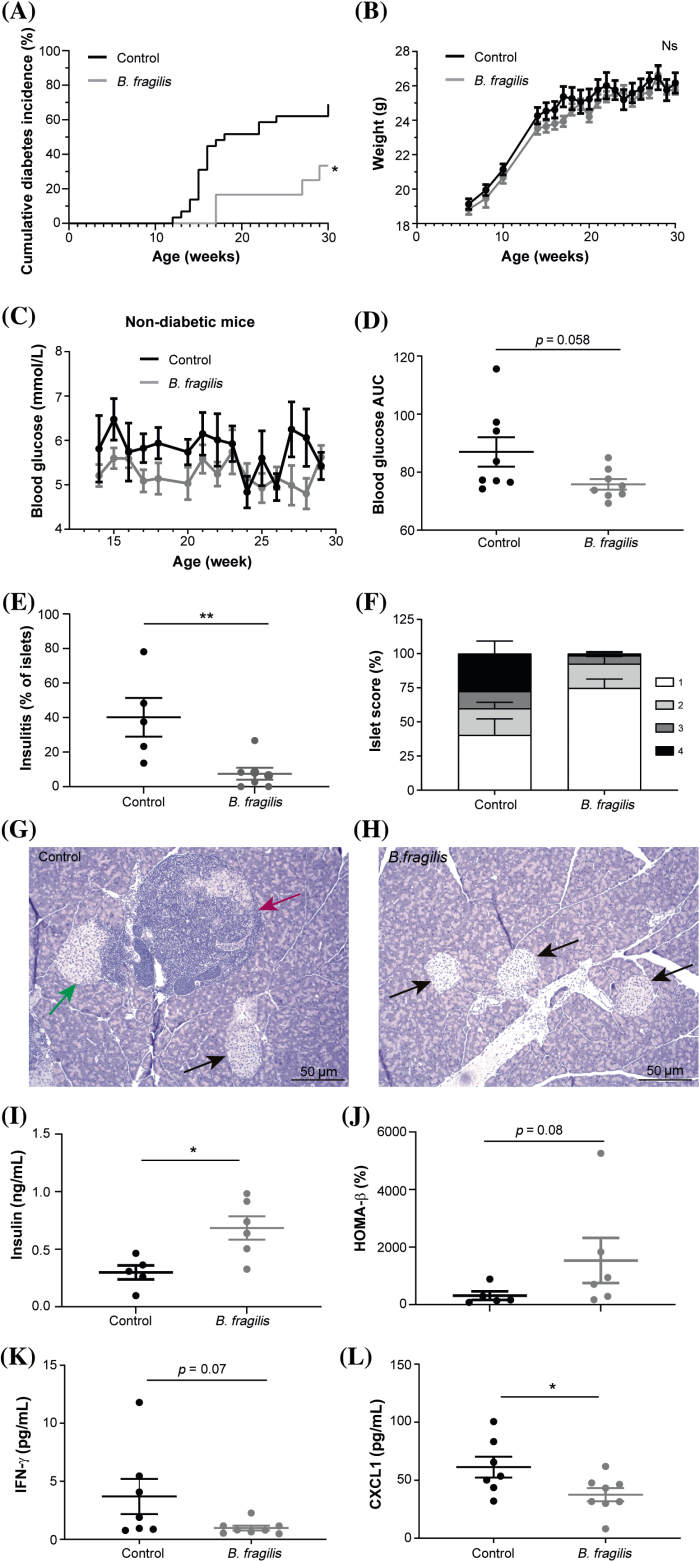
(A) Cumulative diabetes incidence in female nonobese diabetic (NOD) mice treated with *B. fragilis* (BF, *n* = 12) or vehicle (control, C, *n* = 29) from 5 to 8 weeks of age. Comparisons of the survival curves were tested by log‐rank test after 30 weeks of age. (B) Weight and (C) blood glucose levels of the NOD mice during the 30 weeks of diabetes monitoring. (D) Area under the curve (AUC) of the blood glucose levels are shown. Histopathology of pancreatic islets is shown. (E) Percent of islets with insulitis and (F) percent of islets with any given score as indicated is illustrated for nondiabetic 8‐week‐old female NOD mice either treated with BF (*n* = 7) or vehicle C (*n* = 5) since 5 weeks of age. 1, no insulitis; 2, periinsulitis; 3, <50% of the islet is infiltrated by leukocytes; 4, >50% of the islet is infiltrated by leukocytes. (G and H) Representative histological images are shown. Black arrows indicate healthy islets, and green and red arrows represent islets with <50% or >50% of the islet infiltrated with leukocytes respectively. (I) Serum insulin levels and (J) homeostaic model assessment of β cell function (HOMA‐β) values in the same 8‐week‐old nondiabetic female NOD mice. (K) Serum interferon (IFN)‐γ and (L) C‐X‐C motif ligand 1 (CXCL1) concentrations in the nondiabetic 30‐week‐old NOD mice from the incidence study (*n* = 7–8 per group). Mean and SEM are shown. ns, nonsignificant. *p < 0.05, **p < 0.01. *p* values <0.1 are shown.

NOD mice treated with viable *B. fragilis* early in life had delayed diabetes onset time and reduced cumulative diabetes incidence (4 out of 12 became diabetic) compared with control NOD mice (20 out of 29 mice became diabetic) (*p* < 0.05; Figure 1A). The treatment did not affect weight gain of the mice (Figure 1B), but the weekly blood glucose level measurements generally tended (*p* = 0.058) to be lower in the *B. fragilis‐*treated mice throughout the study (Figure 1C–D). At 8 weeks of age, the percentage of pancreatic islets with insulitis was significantly lower in the NOD mice treated with *B. fragilis* (Figure 1E), and more healthy islets were observed compared with the control mice (Figure 1F–H). Moreover, serum insulin levels were significantly higher in the *B. fragilis*‐treated mice compared with the control mice (Figure 1I), and HOMA‐β tended to be higher as well (*p* = 0.08, Figure 1J). Interestingly, the HOMA‐β was lower than the normal range (167–175) for adult humans in three out of five control mice, and in none of the *B. fragilis* mice, supportive of the histological findings. Multiplex analysis of serum of the 30‐week‐old nondiabetic mice showed no major differences in proinflammatory cytokine and chemokine levels except for a tendency of reduced IFN‐γ (*p* = 0.07, Figure 1K) and the chemokine CXCL1 (*p* < 0.05; Figure 1L), the two inflammatory markers usually found elevated in type 1 diabetes patients, reflecting anti‐islet autoimmune activity.^23^, ^24^

### 3.2 The protective effect of *B. fragilis* was solely based on early colonization rather than long‐term changes in the gut microbiome composition

**FIGURE 2:**
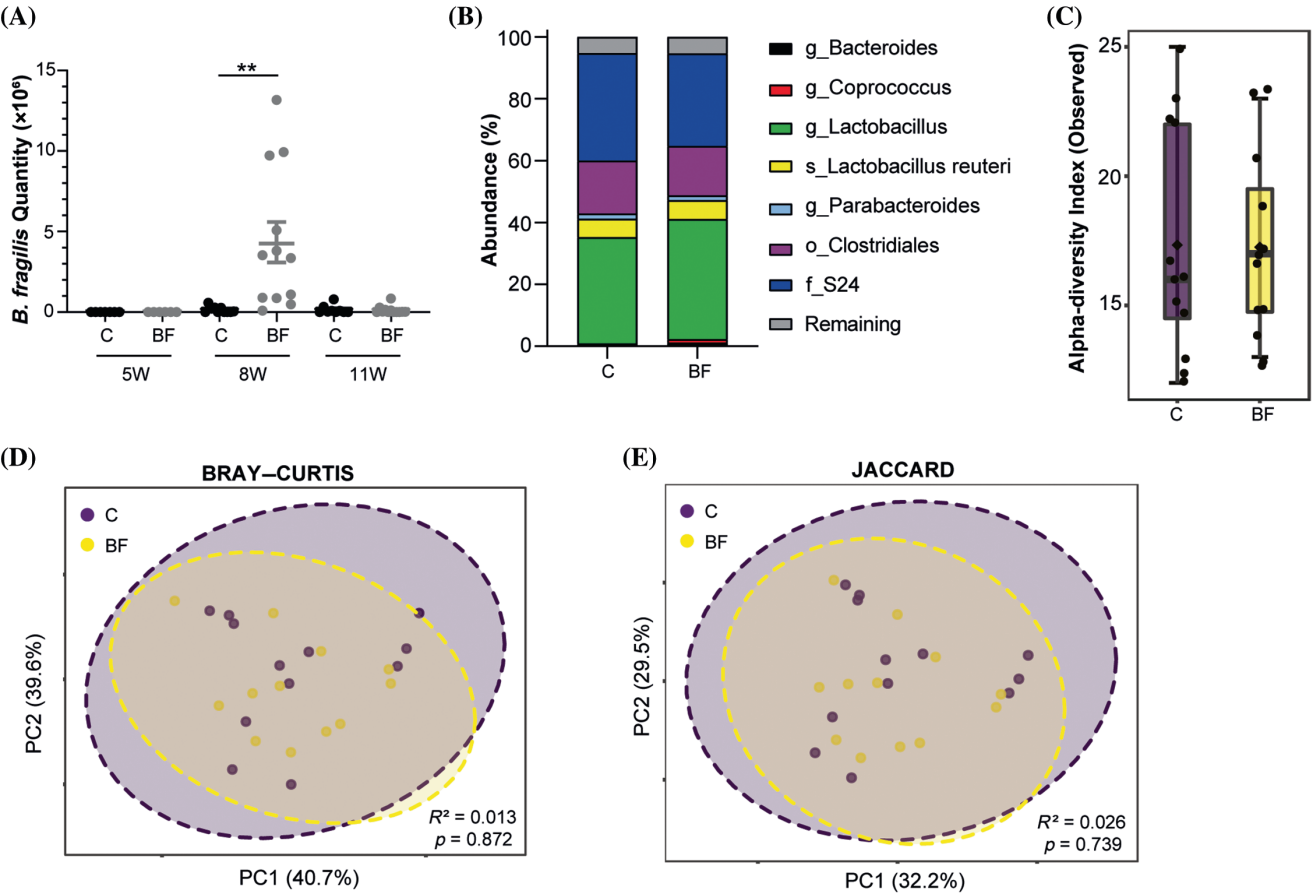
(A) *Bacteroides fragilis* (BF) gene copies/g feces quantified by quantitative polymerase chain reaction (qPCR) in the two groups of BF‐treated (*n* = 12) and vehicle‐treated (control, C, *n* = 12) female NOD mice before (5 weeks of age), during (8 weeks of age), and 3 weeks after (11 weeks of age) treatment. (B) Relative abundance of bacterial taxa in the fecal gut microbiota of the two groups during treatment (8 weeks of age) based on 16S rRNA gene tag‐encoded amplicon sequencing. Only taxa with an abundance above 1% are shown. (C) Alpha‐diversity index (observed) and principal coordinates analysis plot based on Bray–Curtis dissimilarity (D) and Jaccard (E) distance matrices depicting data based on the same 16S rRNA gene tag‐encoded amplicon sequencing as in (B). Error bars represent SEM. **p < 0.01. f, family; g, genus; o, order; s, species. principal components (PC)

To verify colonization of *B. fragilis*, its abundance was determined in feces samples collected before, during, and after treatment. The analysis revealed that all mice in both groups hosted *B. fragilis* at minimal and barely detectable levels in their gut before the treatment started at 5 weeks of age and that the 3 weeks of treatment increased the abundance of *B. fragilis* in the gut >70‐fold compared with the untreated group (Figure 2A). The treatment did not have a long‐lasting colonizing effect, as the bacteria were undetectable in both groups 3 weeks after the treatment had ended. Hence, it was only a short‐term colonization of *B. fragilis* early in life that had long‐lasting effects on the development of type 1 diabetes. The treatment had no additional effect on the gut microbiota composition (Figure 2B). The alpha‐diversity was similar between the groups (Figure 2C), and PCoA plots of 16S RNA gene sequencing of feces samples collected at the end of the treatment period showed no divergent clustering of the groups (Figure 2D,E).

### 3.3 Early‐life exposure to *B. fragilis* products have permanent effects on the regulatory immune system

**FIGURE 3:**
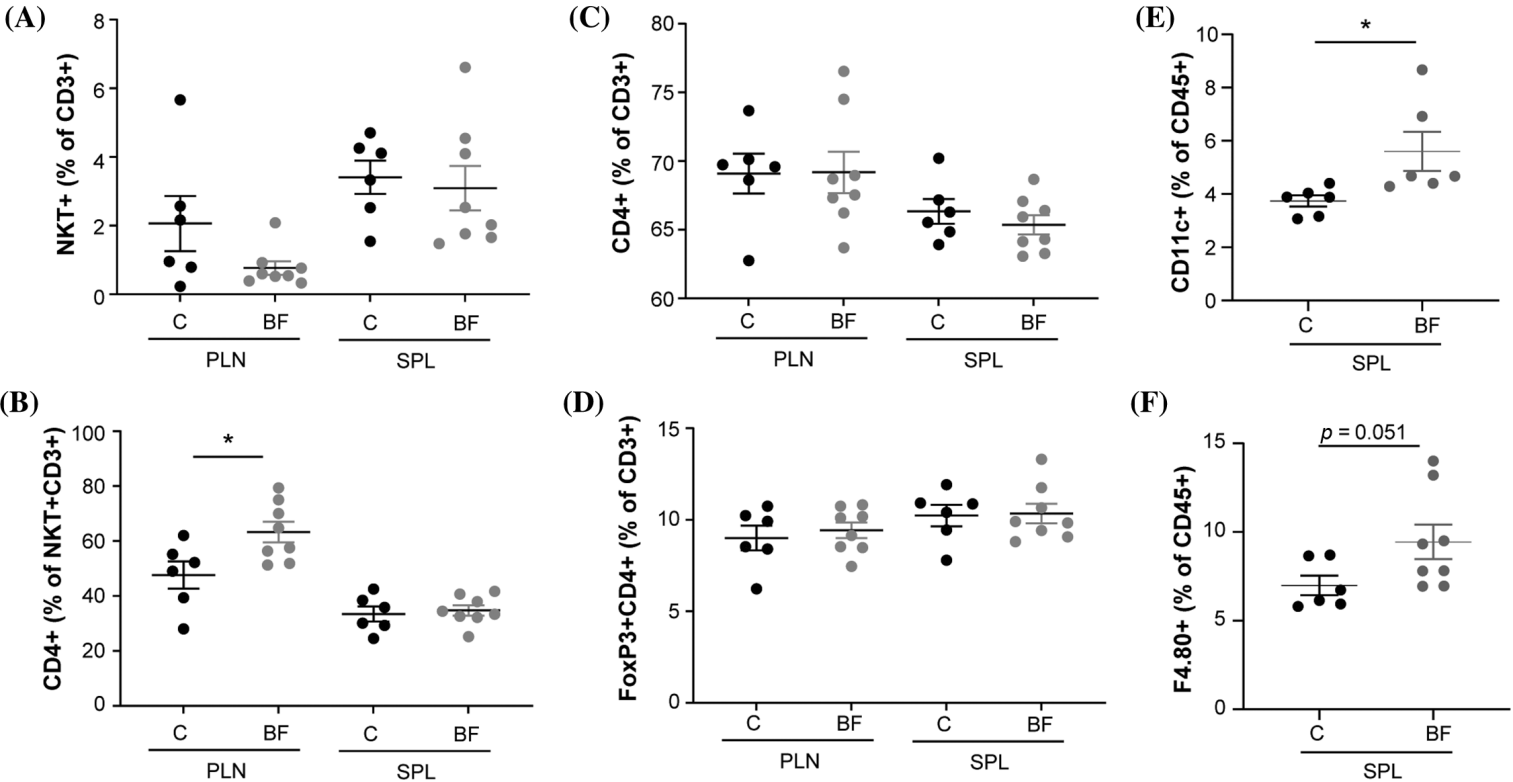
Flow cytometric analysis of (A) the percentage of natural killer T (NKT)^+^ cells out of all T cells (cluster of differentiation (CD)3^+^) and (B) how many of the NKT cells that were positive for CD4^+^, (C) the percentage of T helper cells (CD4^+^) out of all T cells, (D) the percentage of forkhead box P3 (FoxP3)^+^ regulatory T cells out of all T cells, (E) the percentage of dendritic cells (CD11c^+^), and (F) macrophages (F4.80^+^) out of all leukocytes (CD45^+^) isolated from the pancreatic lymph node (PLN) and spleen (SPL) from nondiabetic 30‐week‐old female nonobese diabetic (NOD) mice treated with *B. fragilis* (BF, *n* = 8) or vehicle (control, C, *n* = 6) from 5 to 8 weeks of age. Mean and SEM are shown. NKT, natural killer T. *p < 0.05, *p* values <0.1 are shown.

To investigate whether the *B. fragilis* treatment had any long‐lasting anti‐inflammatory effects on the immune system, the 30‐week‐old nondiabetic mice were euthanized in both groups, and regulatory immune cells were analyzed by flow cytometry. Although the early‐life treatment increased the proportion of CD4^+^ NKT cells in the pancreatic lymph node (Figure 3B), there was no significant effects on the overall percentage of NKT cells (Figure 3A), CD4^+^ T cells (Figure 3C), and FoxP3^+^ regulatory T cells later in life (Figure 3D). The proportion of CD4^+^ NKT cells was similar between the groups in the spleen, but the administration of *B. fragilis* did have minor long‐term systemic effects as well, as the treatment increased the proportion of splenic CD11c^+^ dendritic cells (Figure 3E) and F4.80^+^ macrophages (*p* = 0.051, Figure 3F) compared with the untreated mice.

**FIGURE 4:**
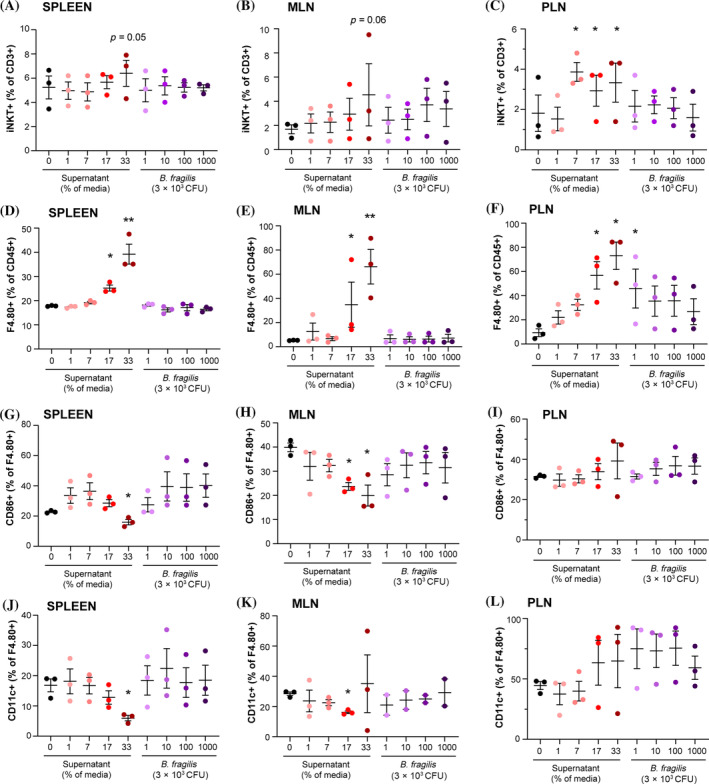
Flow cytometric analysis of leukocytes isolated from spleen (SPL), mesenteric lymph nodes (MLN), and pancreatic lymph nodes (PLN) from six to seven NMRI mice (Taconic) that were cultured ex vivo in complete RPMI culture medium and stimulated overnight with either clean growth media (0% or 33% of the total volume in the well; C, black indicates results pooled from the two clean media control wells), different doses of growth media in which *B. fragilis* was grown in and later separated from (1%, 7%, 17%, 33% of the total volume as indicated) or ultraviolet (UV)‐killed bacteria in four different doses (3 × 10^3^, 3 × 10^4^, 3 × 10^5^, 3 × 10^6^ colony forming units (CFU) per well). The experiment was repeated three times (each dot represents results from six to seven mice pooled together). (A–C) Percentage of invariant NKT (iNKT) cells (alpha‐galactosylceramide [α‐GalCer]‐loaded CD1d tetramer^+^) out of all T cells, (D–F) percentage of macrophages (F4.80^+^) out of all leukocytes, (G–I) percentage of macrophages with costimulatory molecule cluster of differentiation (CD)86^+^, or (J–L) the M1 marker CD11c^+^. Mean and SEM are shown. *p < 0.05, **p < 0.01. *p* values <0.1 are shown.

**FIGURE 5:**
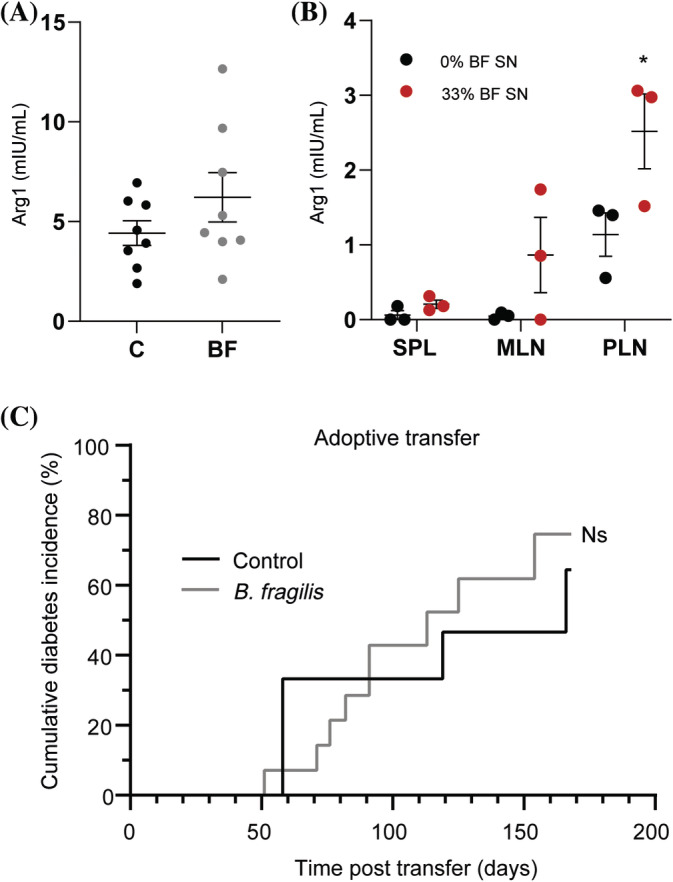
(A) Serum arginase‐1 (Arg1) concentration in nondiabetic 30‐week‐old female nonobese diabetic (NOD) mice treated with *B. fragilis* (BF) or vehicle (control, C) from 5 to 8 weeks of age (*n* = 8 per group). (B) Arg‐1 concentrations in the supernatant (SN) from the ex vivo organ cell culture study described in Figure 4, from the clean (0% BF SN) or BF growth media (33% BF SN) supplemented wells with highest doses. (C) Splenocytes from 8‐week‐old female NOD mice treated with BF or vehicle since 5 weeks of age were adoptively transferred to two groups of 8‐week‐old female nonobese diabetic/severe combined immunodeficiency (NOD/SCID) mice. Cumulative diabetes incidence 24 weeks posttransfer is shown. Mean and SEM are shown. MLN, mesenteric lymph nodes; ns, nonsignificant; PLN, pancreatic lymph nodes; SPL, spleen. *p < 0.05.

Next, we sought to clarify whether the local effect on NKT cells and systemic effects on antigen‐presenting cells were mediated directly by the bacteria or their products (α‐GalCer). We therefore stimulated both pancreatic and mesenteric lymph nodes, as well as splenocytes from NMRI mice, with either UV‐killed *B. fragilis* no longer able to produce α‐GalCer or with varying doses of the culture medium that the commercially available α‐GalCer‐producing *B. fragilis* had been grown in. We were, as expected, not able to detect any significant effects on iNKT cells of the UV‐killed bacteria themselves, but we found that their culture medium upregulated iNKT cells overnight in a dose‐dependent manner. Only the highest dose of *B. fragilis* culture medium was able to induce a slight increase in the spleen (*p* = 0.05, Figure 4A) and mesenteric lymph node (*p* = 0.06, Figure 4B). However, in accordance with the flow cytometry results from the NOD mice, the effect was much more pronounced locally in the pancreatic lymph node, where the induction of iNKT cells was already observed at lower doses of culture medium (Figure 4C). The high proportion of macrophages was also induced ex vivo by the *B. fragilis* culture medium, an effect that was observed in all tested organs at the highest doses of culture medium (Figure 4D–F). There was a profound upregulation of macrophages without activation marker CD86 and M1 marker CD11c in all *B. fragilis* medium‐stimulated lymph nodes and splenocytes compared with the control cells treated with clean medium (Figure 4G–L). We therefore suspected that the upregulation was based primarily on tolerogenic M2 macrophages. To verify this, the M2 marker arginase was measured in the serum of treated mice, as well as in the cell supernatant 24 h, after adding the *B. fragilis* culture medium. Despite a tendency, the arginase concentration was not significantly higher in the serum of the treated NOD mice (Figure 5A), but it was higher in the supernatant after addition of the *B. fragilis* culture medium (Figure 5B), verifying the flow cytometry results that indicated an induced shift from M1 to M2 macrophages by *B. fragilis* derived products.

To determine whether the systemic anti‐inflammatory effect on the splenocytes was antidiabetogenic, splenocytes isolated from 8‐week‐old nondiabetic NOD mice either treated or not with *B. fragilis* was adoptively transferred into NOD/SCID mice. Blood glucose levels were monitored in the recipient mice for 25 weeks, but the incidence and onset of diabetes did not differ significantly between the two groups (Figure 5C). We therefore concluded that the effect of *B. fragilis* on type 1 diabetes was not due to a systemic impact on the immune system.

### 3.4 Gut barrier function and immune activation in the gut were unaffected by *B. fragilis*

**FIGURE 6:**
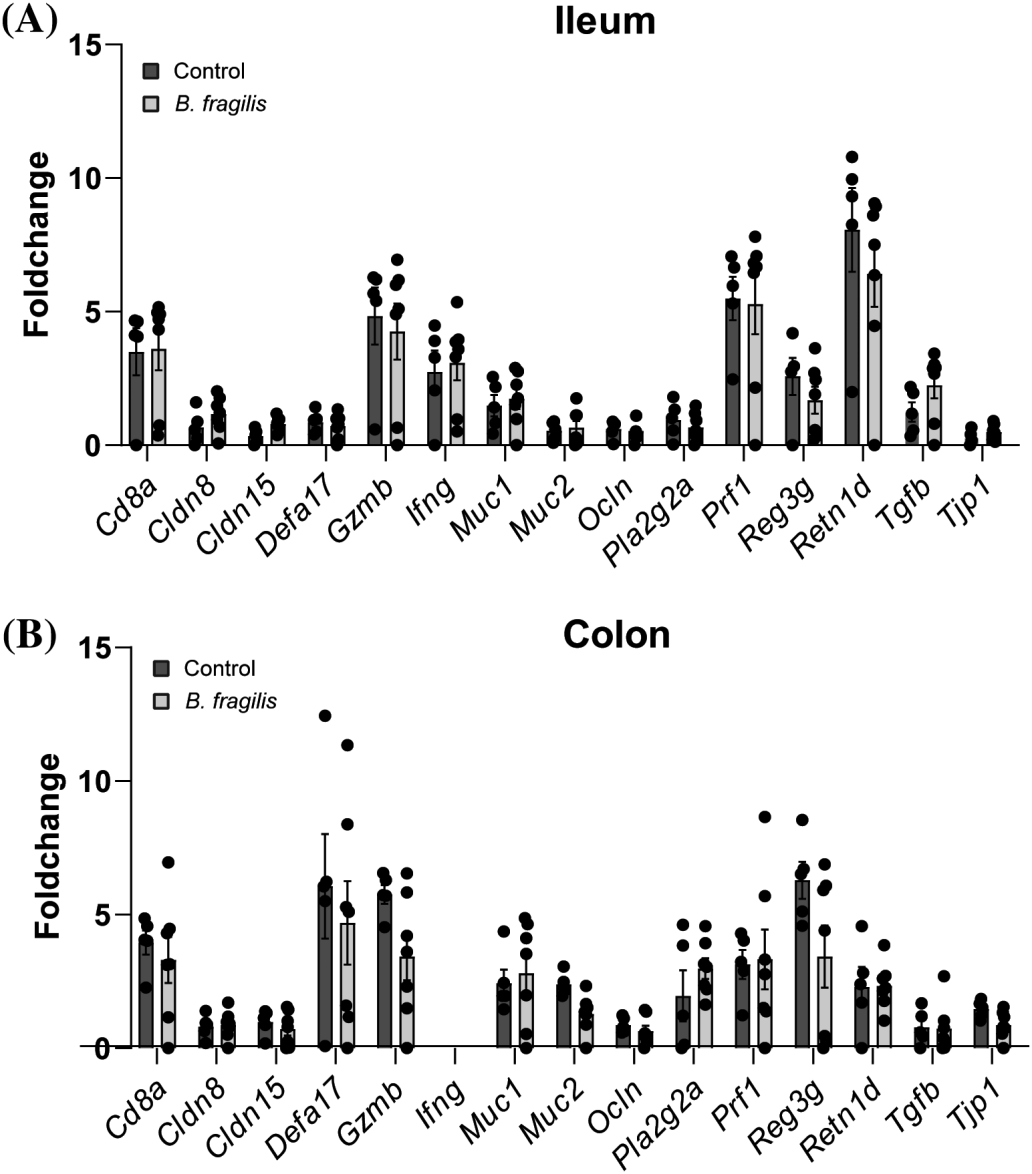
Relative gene expression of *Cd8a*, *Cldn8*, *Cldn15*, *Defa17*, *Gzmb*, *Ifng*, *Muc1*, *Muc2*, *Ocln*, *Pla2g2a*, *Prf1*, *Retn1d*, *Tgfb*, and *Tjp1* in (A) ileum and (B) colon biopsies sampled at 8 weeks of age from female nondiabetic nonobese diabetic (NOD) mice after 3 weeks of oral treatment with either *B. fragilis* (light gray bars, *n* = 5) or vehicle (dark gray bars, *n* = 7). Error bars represent SEM.

Impaired gut barrier function has previously been associated with increased intestinal immune activation and diabetes incidence in NOD mice.^25^ Gene expression of barrier related and inflammatory markers in ileum and colon of the 8‐week‐old donor mice from the adoptive transfer experiment were therefore analyzed by qPCR. None of the genes were differentially expressed between the *B. fragilis*‐ and vehicle‐treated mice after correcting for multiple testing (Figure 6A,B). *Ifng* was only expressed in ileum. Only *Muc2* was significantly reduced in the colon in the *B. fragilis* group before adjusting for multiple testing, and hence, we could conclude that improved gut barrier function was not involved in the reduced diabetes incidence.

## 4 DISCUSSION

Microbiome perturbation is a well‐known environmental regulator of autoimmune destruction of the pancreas,^26^, ^27^, ^28^ although only a handful of bacteria have been proven to reduce disease progression in animal models and suggested as probiotics for management of type 1 diabetes.^29^ Here, we demonstrate that a common gut commensal *B. fragilis* has protective effects against type 1 diabetes upon colonization early in life. *B. fragilis* is a common member of the adult intestinal microbiome constituting up to 1%–2% of cultured fecal bacteria and tends to be even more prevalent in the infant gut.^30^ Persistent colonization failed in the young NOD mice, but the transient effect was sufficient to mitigate diabetes development. Even earlier colonization in the postnatal period may prove to be more effective for long‐term colonization and health benefits in the host.^31^ The nutrient composition in human diet strongly affects the abundance of *B. fragilis* after the first year of life as *B. fragilis* metabolizes polysaccharides and oligosaccharides.^32^ Favorable conditions to allow for its persistence after probiotic administration may therefore be more easily achieved by supplementing the diet with oligosaccharides. In support of this notion, several *Bacteroides* species were upregulated in NOD mice when fed xylooligosaccharides‐supplemented diet early in life.^21^

*B. fragilis* is already acknowledged as a key immune regulator orchestrating dual immunomodulatory effects in its host. While studies have established *B. fragilis* as an opportunistic pathogen involved in systemic inflammation and infections in patients with compromised gut barrier function, it has also been suggested as a next‐generation probiotic due to its profound anti‐inflammatory and health benefits in a range of diseases.^33^ Depending on the administration route or the state of the intestinal barrier, the cell membrane component polysaccharide A from heat‐killed *B. fragilis* have been found to, for example, either induce or suppress autoimmunity in NOD mice in a toll‐like receptor 2‐dependent manner.^34^ However, in addition to the well‐characterized biological activities of polysaccharide A,^35^, ^36^, ^37^ *B. fragilis* also produces the immunogenic glycosphingolipid α‐GalCer that is the prototypical agonist of CD1d‐restricted iNKT cells.^5^ In the current study, *B. fragilis* induced CD4^+^NKT cells in the pancreatic lymph nodes, but the inducible costimulator (ICOS)/NKT cell marker used to identify the cells also targets type II NKT cells.^38^ Type II NKT cells are distinguished from iNKT cells by a broader T‐cell repertoire and their CD1d‐restricted self‐lipid reactivity including mammalian β‐glycosphingolipid sulfatides.^39^ Activation of type II NKT cells following sulfatide treatment has been shown to be immunosuppressive as well in autoimmune diabetes.^40^ Especially, the CD4^+^ subset high in ICOS expression, but not CD4/CD8 double negative type II NKT cells, can downregulate diabetogenic CD4^+^ BDC2.5 NOD T cells in adoptive transfer experiments.^38^

To verify the proliferation of the type I iNKT cell subset by *B. fragilis*, leukocytes isolated from both systemic and local lymphoid tissues were stimulated ex vivo with *B. fragilis* culture media, which increased the proportion of α‐GalCer‐loaded CD1d tetramer‐stained iNKT cells in especially the pancreatic lymph node cells, and inherently demonstrated α‐GalCer activity in the viable bacteria. Also in the NOD mice, the induction of CD4^+^ NKT cells was only observed in the draining pancreatic lymph node, and the missing systemic effect was confirmed by adoptive transfer of splenocytes from *B. fragilis*‐treated NOD mice that was unable to transfer protection against diabetes. UV‐killed *B. fragilis* failed to stimulate iNKT cells in a similar manner as the culture media from viable bacteria, likely due to the lack of α‐GalCer in UV‐killed bacteria, which suggested that *B. fragilis* can mitigate diabetes development in NOD mice in more than one way. In addition to the polysaccharide A‐mediated protection, viable α‐GalCer‐producing *B. fragilis* can induce iNKT cells with the ability to regulate autoimmunity in NOD mice. In particular, the CD4^+^ iNKT subpopulation has a stronger immunoregulatory role compared with the CD4^−^ iNKT cells that tend to produce more Th1‐type cytokines and possess strong cytolytic capacity.^41^, ^42^ Quantitative deficiencies of CD4^+^ iNKT cells has been observed in type 1 diabetes patients,^43^ and while transfer of CD4^−^ iNKT cells exacerbated the incidence of diabetes in transgenic NOD mouse models, the CD4^+^ iNKT cells prevented diabetes development,^44^ demonstrating its importance in regulating the risk of developing type 1 diabetes.

α‐GalCer activation of iNKT cells promotes anti‐inflammatory CD11c‐negative M2 macrophage polarization^45^ and induces tolerogenic dendritic cells in the pancreatic lymph nodes of NOD mice with reduced expression of costimulatory receptors.^46^ Similar induction of M2 macrophages with reduced expression of CD86 was observed in the spleen and mesenteric lymph nodes of *B. fragilis* culture media stimulated lymphoid cells, supporting the notion that α‐GalCer activation of iNKT by *B. fragilis* can confer protection in NOD mice. In contrast, no difference in tolerogenic dendritic cells was observed, and M2 macrophage polarization was only observed in the spleen and mesenteric lymph node, whereas the iNKT cell induction was most pronounced in the pancreatic lymph node. As the adoptive transfer of systemic splenocytes failed to induce protection in the NOD/SCID mice, other mechanisms related to the more local gut‐pancreas environment may be involved which need further investigation. For example, tolerogenic antigen‐presenting cells promote migration of regulatory T cells into pancreatic islets and favor anergy of autoreactive T cells as previously shown,^47^, ^48^ but it is important to stress that iNKT cells can mediate their regulatory functions by several mechanisms.^49^ Further understanding of the biology behind the regulatory capacity of *B. fragilis* on iNKT cell activation, as well as the most beneficial timing for its administration, is imperative for its future in treating iNKT cell imbalance. In light of the current study, and considering the importance of iNKT cells in type 1 diabetes, α‐GalCer‐producing *B. fragilis* might very well be a next‐generation probiotic for the children at risk of developing type 1 diabetes.

## AUTHOR CONTRIBUTIONS

CHFH, DSN, AKH, and KB contributed to conception and design of the studies and interpretation of data. CHFH, DJ, and LFZ contributed to acquisition of data and analysis. CHFH and KB drafted the manuscript, and all authors revised it critically and made final approval of the submitted versions.

## FUNDING INFORMATION

The study was partly funded by “Sigrid Rigmor Morans Mindefond.”

## CONFLICT OF INTEREST STATEMENT

The authors declare no conflicts of interest.

## Supporting information


**Figure S1.** Representative plots and gating strategy for flow cytometric analysis of splenic (A) CD4+ NKT cells and regulatory T cells, and (B) macrophages and dendritic cells. The gates are based on fluorescence minus one (FMO) control stains (not shown).

## Data Availability

Data are available on request from the authors, and will be published in an open online repository before publication.
