# Supplementary figures and images for: Probiotic treatment with viable α‐galactosylceramide‐producing Bacteroides fragilis reduces diabetes incidence in female nonobese diabetic mice
Source: J Diabetes. 2024 Aug 13;16(8):e13593. doi: 10.1111/1753-0407.13593 (PMC11320754; doi:10.1111/1753-0407.13593)

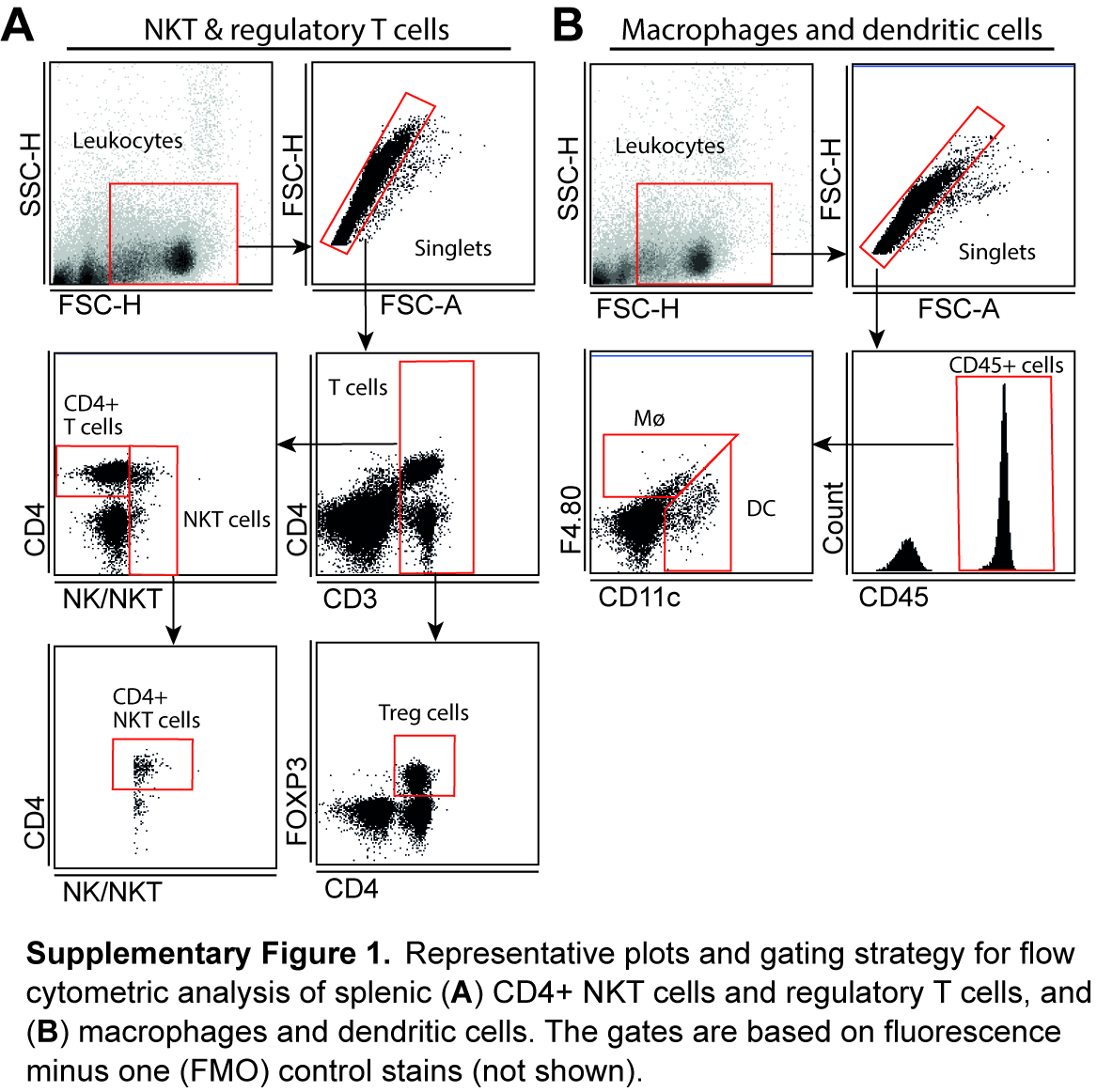

Supplement: Supplementary file 1 — Figure S1. Representative plots and gating strategy for flow cytometric analysis of splenic (A) CD4+ NKT cells and regulatory T cells, and (B) macrophages and dendritic cells. The gates are based on fluorescence minus one (FMO) control stains (not shown). [file JDB-16-e13593-s001.tif]
